# How do Funeral Practices Impact Bereaved Relatives' Mental Health, Grief and Bereavement? A Mixed Methods Review with Implications for COVID-19

**DOI:** 10.1177/0030222820941296

**Published:** 2020-07-08

**Authors:** Alexander Burrell, Lucy E. Selman

**Affiliations:** 1 North Bristol NHS Trust, Bristol, UK; 2 Population Health Sciences, Bristol Medical School, University of Bristol, UK

**Keywords:** bereavement, grief, funerals, burial, mental health

## Abstract

Those who are bereaved during the current COVID-19 pandemic are subject to restrictions on funeral sizes and practices. We conducted a rapid review synthesising the quantitative and qualitative evidence regarding the effect of funeral practices on bereaved relatives’ mental health and bereavement outcomes. Searches of MEDLINE, PsycINFO, KSR Evidence, and COVID-related resources were conducted. 805 records were screened; 17 studies of variable quality were included. Current evidence regarding the effect of funeral practices on bereaved relatives’ mental health and bereavement outcomes is inconclusive. Five observational studies found benefits from funeral participation while six did not. However, qualitative research provides additional insight: the benefit of after-death rituals including funerals depends on the ability of the bereaved to shape those rituals and say goodbye in a way which is meaningful for them. Findings highlight the important role of funeral officiants during the pandemic. Research is needed to better understand the experiences and sequalae of grief and bereavement during COVID-19.

## Introduction

The coronavirus disease 2019 (COVID-19) has caused 383,000 deaths globally as of June 4th 2020, with c.106,000 deaths in the USA and 40,000 confirmed COVID-19 deaths in the UK (World Health Organization, 2020). In addition, there is an as-yet undetermined number of excess deaths associated with the pandemic; in the UK, overall figures since the start of the COVID-19 crisis are a fifth higher than usual (Office of National Statistics, 2020). To attempt to reduce infection rates and therefore mortality due to COVID-19, governments have implemented public health measures designed to reduce interactions between people. This includes restrictions on the number of mourners permitted to attend funerals as well as minimising interactions with the deceased during ceremonies, which affect all of those bereaved during the current crisis (Centers for Disease Control and Prevention, 2020; Public Health England, 2020). In the UK, for example, guidance for the foreseeable future states that the number of mourners is to be kept as low as possible to ensure a safe distance of at least 2 metres can be maintained between individuals (UK Government, 2020). Alongside funeral staff and an officiant (usually a non-denominational celebrant or a faith representative), permitted attendees are members of the deceased’s household, close family members, or, if these are unable to attend, close friends. While mourners unwell with symptoms of COVID-19 should not attend, those who are extremely clinically vulnerable can decide to do so despite the risk. Funeral venue managers are instructed to set caps on numbers to ensure social distancing can be maintained, consider how to manage the flow of attendees in and out of venues, ensure ventilation and regular disinfection of surfaces, and provide adequate handwashing stations. To ensure that organisations managing funerals are able to cope with the increased number of deaths, it is requested that funerals are not delayed.

In practice, these formal requirements mean mourners lack expressions of physical comfort (through hugs, handshakes or sitting next to each other during the funeral), are not able to touch the coffin, cannot hold a reception after the funeral to socialise and may not feel that they have said the farewells they would have wished for. The measures in place due to COVID-19 mean that funerals will most likely not resemble what the bereaved or the deceased would have wanted, and many mourners will be unable to attend funerals in person. Funerals are a fundamental component of cultural and religious mourning systems: they facilitate the offering of social and psychological support to the bereaved, and afford an opportunity to convey love and respect for the deceased (O’Rourke et al., 2011). It is consequently possible that being unable to participate in funerals, rituals, and ceremonies will have a detrimental effect on the bereaved, affecting their mental health and ability to cope with or process their grief. This rapid review therefore aims to synthesize evidence on the impact of funeral practices on bereaved friends and relatives’ mental health and experience of bereavement. We then consider implications for the COVID-19 pandemic.

## Methods

### Design

Rapid systematic review according to Preferred Reporting Items for Systematic Reviews and Meta-Analyses (PRISMA) guidelines (Online Appendix 1).

### Inclusion/Exclusion Criteria

- Population – bereaved family members/friends
- Intervention – funeral practices and rituals including burial rites and ceremonies
- Context – N/A
- Outcomes – mental health and bereavement outcomes assessed quantitatively (including but not limited to depression, prolonged grief disorder, PTSD symptoms, anxiety, grief intensity), and/or qualitative findings regarding experiences of grief and bereavement

#### Included

- Original quantitative or qualitative studies or systematic reviews of the mental health or bereavement outcomes of bereaved families/friends, including children, in relation to funeral practices

#### Excluded

- Non-English language study reports
- Studies of health or social care staff or funeral directors
- Studies related to bereavement after a pet’s death
- Studies related to stillbirth, miscarriage, neonatal death or death of a child during the first year of life
- Studies of bereaved people not examining mental health, bereavement outcomes or grief experiences
- Opinion pieces, narrative reviews, dissertations, conference abstracts

### Search Strategy

We searched for relevant articles using MEDLINE (Ovid) and PsycINFO databases with no date limits. We also searched KSR Evidence, Rayyan “COVID-19 Open Research Dataset” and other COVID-related resources. The search strategy comprised terms for funerals, bereavement, mourning, grief, and mental health outcomes (Table 1).

**Table 1.** Search Strategy and Terms.

Medline and PsycInfo	Medline search:1. Bereavement/ (exp) 2. Grief/ (exp) 3. Bereave*.tw4. Griev*.tw5. Mourn*.tw6. Mental Health/ (exp) 7. Mental Disorders/ (exp) 8. 1 or 2 or 3 or 4 or 5 or 6 or 79. Funeral rites/ (Exp) 10. Burial/ (Exp) 11. Cremation/ (Exp) 12. Embalming/ (Exp) 13. Funeral.tw14. Burial.tw15. 9 or 10 or 11 or 12 or 13 or 1416. 8 and 15 PsycInfo search: 1. Bereavement/2. Grief/3. Bereave*.tw. 4. Griev*.tw. 5. Mourn*.tw. 6. Mental health/7. Mental Disorders/ 8. Death rites9. Funeral.tw. 10. Burial.tw. 11. 1 or 2 or 3 or 4 or 5 or 6 or 712. 8 or 9 or 1013. 11 and 12
KSR Evidence	1. “Bereavement” in All text2. “Grief” in All text 3. Bereave* in All text 4. Griev* in All text5. Mourn* in All text6. “Mental health” in All text7. “Mental disorders” in All text8. #1 or #2 or #3 or #4 or #5 or #6 or #79. “Funeral rites” in All text 10. “Burial” in All text 11. “Cremation” in All text12. “Embalming” in All text13. Funeral* in All text 14. Burial* in All text15. #9 or #10 or #11 or #12 or #13 or #1416. #8 and #15
Rayyan “COVID-19 Open Research Dataset”	Screened articles retrieved with any of the following search terms for title, abstract or author: 1. Bereavement2. Grief3. Funeral4. Mourn5. Burial6. Religion
CEBM, University of Oxfordhttps://www.cebm.net/covid-19/	“bereavement”, “grief”, “funeral”, “mourn”, “burial”, or “religion”
Evidence aidhttps://www.evidenceaid.org/coronavirus-resources/	
Cochrane Methodology Review GroupInfection control and prevention: https://www.cochranelibrary.com/collections/doi/SC000040/fullEvidence relative to critical care: https://www.cochranelibrary.com/collections/doi/SC000039/full	
Department of Health and Social Care Reviews Facilityhttp://eppi.ioe.ac.uk/COVID19_MAP/covid_map_v3.html	
UCSF COVID19 papershttps://ucsf.app.box.com/s/2laxq0v00zg2ope9jppsqtnv1mtxd52z	
PHE Knowledge and Library Serviceshttps://phelibrary.koha-ptfs.co.uk/coronavirusinformation/	
WHO Global Research COVID19 databasehttps://www.who.int/emergencies/diseases/novel-coronavirus-2019/global-research-on-novel-coronavirus-2019-ncov	
CDC COVID19 guidancehttps://www.cdc.gov/coronavirus/2019-ncov/hcp/index.html	
Cochrane COVID-19 Study Registerhttps://bit.ly/2x7vwGX	

### Study Selection

One author (L. S.) completed the MEDLINE (Ovid) and PsycINFO searches and deduplicated records. The other author (A. B.) completed the remaining searches. Article titles and abstracts were screened in EndNote (version X9, Clarivate Analytics, Philadelphia, PA) by A. B. The full-texts of articles not excluded on the basis of title and abstract were independently dual-screened by A. B. and L. S.

### Data Extraction

Data were extracted into a bespoke results table. A. B. extracted the data, with extraction reviewed by L. S.

### Analysis

Study findings are presented in Table 2 and synthesised narratively. Study quality and risk of bias were assessed using the Quality Assessment Tool for Observational Cohort and Cross-Sectional Studies [https://www.nhlbi.nih.gov/health-topics/study-quality-assessment-tools] and the Critical Appraisal Skills Programme Qualitative Checklist [https://casp-uk.net/casp-tools-checklists/] for quantitative and qualitative studies respectively. Quality appraisal was performed by A. B. and reviewed by L. S. with disagreements discussed and resolved. Quality assessments were tabulated, with limitations summarised and integrated into the narrative.

**Table 2.** Results Table.

Author (year) *Country*	Study design	Number	Participant demographics	Summary of results	Study limitations
Bolton & Camp (1987) *U.S.*	**Study design:** observational cross-sectional quantitative survey**Population:** widowed persons selected at random from previous clients of Widowed and Family Grief Counselling Program**Intervention:** self-reported number of pre-, during-, and post-funeral rituals**Outcome measures:** two measures of grief adjustment – *Affect-Balance Scale and Attitude Inventory*	50	Mean age 55.6 years94% femaleAverage of 6 years since death of spouse	“No statistically significant degree of association for the major variables were produced” i.e. no statistically significant relationship between number of pre-, during-, and post-funeral rituals and grief adjustment measures. No reporting of interpretive statistical data to support this “Subscales of the Attitude Inventory (usefulness, health, happiness, and financial) were clearly related to post-funeral rituals such as sorting personal effects, removing the wedding ring, visits to the grave side, and the disposal of personal effects”. No reporting of interpretive statistical data to support this	Random selection process not specified, small sample size, almost all female sample, time since bereavement variable, interpretive statistical data on primary outcome and subanalysis not reported
Doka (1985) *U.S.*	**Study design:** observational cross-sectional mixed methods – quantitative survey with qualitative interview**Population:** survivors who had primary responsibility for arranging funeral rituals of a death that occurred 12–18 months prior to the onset of the study obtained from referrals from students, clergy, senior citizens groups and funeral homes**Intervention:** self-reported participation in planning and conducting funeral rituals**Outcome measures:** adjustment to death using a modification of the *Carey Adjustment Scale*	50	100% white middle to upper class76% femaleAge: 12% 18–35 years, 45% 36–60 years, 28% ≥61 years60% Protestant, 32% Catholic, 8% Jewish	“There were no significant differences between involvement [in planning and conducting of funeral rituals] and grief adjustment a year later” Chi-Square value for planning of funeral rituals and grief adjustment: 1.09, p-value not reportedChi-Square value for participation in conducting funeral rituals and grief adjustment: 0.94, p-value not reportedCatholics were significantly less likely to report participation in planning funeral rituals that Jewish or Protestant respondents (Chi-square value: 6.75, p < 0.01) *Participating in planning funeral arrangements:* 57% of those who participated in planning felt it helped them with their grief, while another 28% were unsure. Some felt the busyness itself was worthwhile. Others found that the intensive involvement with caregivers was comforting. Others derived comfort from personalizing and being part of the service. 2 participants found that involvement reaffirmed their own abilities and worth. The 15 percent that did not define the planning process as helpful were not negative toward participation but tended to look on the task as a routine duty associated with funerals. Half of the 20% who did not assist in planning defined themselves as emotionally incapable of doing so at the time. Only 20% of the sample reported actual participation by themselves or other family members in the funeral service itself. All but one respondent reported that this too eased grief.	Small sample size, ethnically, socially and religiously homogenous sample, predominantly female sample
Fristad et al. (2001) *U.S.*	**Study design:** observational prospective cohort quantitative survey**Population:** parent-bereaved children aged 5–17 years recruited from obituaries from local newspapers and contact with local funeral homes for the Grief Research Study**Intervention:** participation in the visitation, funeral and burial using *The Funeral Questionnaire – Child and Parent Forms***Outcome measures:** grief measured using *The Grief Interview – Child and Parent Forms*, depressive symptomology measured using *The Child’s Depression Rating Scale – Revised* and *Diagnostic Interview for Depression in Children and Adolescents*, symptoms of 16 psychiatric disorders using *The Diagnostic Interview for Children and Adolescents*, and overall psychiatric symptomology using *BAMO scale*. Outcomes measured at 1, 6, 13- and 25-months post-death	318	59% age 5–12 years, 41% age 13–17 years98% Caucasian	“Nearly all children whose families had visitations, funerals, and burials attended. Thus, comparisons could not be made between those children who attended and those children whose families had the ritual but did not attend … Therefore comparisons were made between 258 children who attended a visitation and the 38 children whose families did not have a visitation” No differences were found between groups at 1 and 6 months post-parental deathBy 13 months post-loss, overall symptomology was 50% lower for children who did versus did not attend the visitation ( (0.6 ± 0.8 vs. 1.2 ± 1.5; t 2.26, df 37.7, p <.03) as well as depressive symptom severity (22.3 ± 7.7 vs. 30.6 ± 13.4; t 2.36, df 15.4, p < .03) By 25 months post-loss, those children who had attended the visitation had fewer PTSD symptoms than those who did not (0.4 ± 0.7 vs. 0.7 ± 0.8; t 2.08, df 188, p < .05).	Ethnically homogenous sample, analysis changed to suit participants not pre-specified, clinical significance not considered
Gamino et al. (2000) *U.S.*	**Study design:** observational cross-sectional mixed methods – quantitative survey with qualitative interview**Population:** bereaved individuals participating in the Scott & White grief study: recruited from outpatient psychiatry clinic; family and friends sent a condolence letter after loved one died in hospital; self-help/grief support groups; personal contact from investigators**Intervention:** self-reported participation in funeral planning, funeral/burial service attendance, perceptions of funeral experience, whether funeral service was described as comforting, any adverse events reported in connection with funeral/burial rites**Outcome measures:** grief symptomology measured by *Grief Experience Inventory*	74	Mean age 50.7 years78.4% female91.9% white, 4.1%African, 4.1% Hispanic4.1% no religion, 28.4% mainline Protestant, 44.6% conservative protestant, 1.4% Pentecostal, 18.9% Catholic, 2.7% Jewish	Mourners who described funeral/burial services as “comforting” reported significantly less overall grief (F = 5.33, p = .01) and subscales of social isolation (F = 7.28, p = 0.005), despair (F = 5.34, p = 0.01), anger/hostility (F = 4.04, p = 0.02) and guilt (F = 2.93, p = 0.05) “Nearly every death was followed by a funeral or memorial service … Among those mourners with the opportunity to attend services, almost all chose to do so. Therefore, no meaningful statistical distinction could be drawn between [those who attended and those who did not]”. Those who participated in planning the funeral reported significantly lower depersonalisation (F = 4.10, p = 0.001) and social isolation (F = 2.91, p = 0.05) than those who did notThose who experienced adverse events (e.g. conflicts among survivors, discrepancies between the expressed wishes of the decedentand the preferences of the survivors, issues with cremation, state of the body, problems with the funeral home, problems with the minister, financial problems) during the funeral service had significantly higher overall grief (F = 3.45, p = 0.05), and subscales of somatization (F = 10.73, p = 0.001), loss of control (F = 4.84, p = 0.02) and depersonalization (F = 2.89, p = 0.05)	Ethnically homogenous sample, predominantly female sample, time since bereavement variable, clinical significance not considered
Grabowski & Frantz (1993) *U.S.*	**Study design:** observational cross-sectional quantitative survey**Population:** volunteer participants of Latino and Anglo origin who had experienced the death of a relative, friend or acquaintance **Intervention:** funeral attendance or novena participation (nine-day post-funeral practice involving prayer and support that generally takes place in the home of the bereaved) **Outcome measures:** intensity of grief reactions measured by *Texas Revised Inventory of Grief (TRIG) – Part I measures adjustment to past life event i.e. death, Part II measures intensity of present feelings of grief over loss of a loved one*	50 Latino and 50 Anglo participants	Of all 100 participants, 95% Roman Catholic69% femaleMean age 47 years	No significant difference in grief intensity between those who did and did not attend the funeral in Latino and Anglo samples (F = 0.5, p value not reported) In the Latino sample there was no significant difference in grief intensity between those who had and had not participated in a novena (F = 1.11, p value not reported) In the Latino sample who had attended a novena, there was no significant correlation between their self-report of helpfulness of a novena and grief intensity (t = 01.506 for Part I and t = −0.932 for Part II of TRIG)	Religiously homogenous sample, volunteer sample, TRIG in English only, time since bereavement variable
Kissane, et al. (1997) *Australia*	**Study design:** observational prospective cohort quantitative survey**Population:** bereaved spouses with one or more children aged 12 years or older of a relative who died from cancer when aged 40-65 years**Intervention:** use of mourning rituals and “relevant aspects of the death and funeral [including] saying goodbye and viewing the corpse” using an interview with the bereaved spouse including qualitative and quantitative components on four-point Likert scales**Outcome measures:** grief measured by *Bereavement Phenomenology Questionnaire (BPQ)*, psychological morbidity using cognitive items of *Beck Depression Inventory* (*BDI)* and *Brief Symptom Inventory (BSI),* social functioning using *Social Adjustment Scale (SAS),* at 6 weeks (T1), 6 months (T2) and 13 months (T3) following the death	115 at T1, 104 at T2, 100 at T3	Mean age 55.9 years53% female66% Australian, 11% English, 7% Eastern European, 5% Italian, 4% Irish, 2% Asian, 1% Greek, 4% other85% Christian, 3.5% Jewish, 8% no religion	Not viewing the body of the deceased correlated with BDI i.e. more depressive symptoms at T1 (Pearson correlation 0.3297, p < 0.001) and negatively correlated with BPQ i.e. more grief intensity T1 (Pearson correlation −0.3905, p < 0.01). However due to small numbers this variable was not included in best subset regression analyses. Saying goodbye as wished correlated with SAS i.e. better social adjustment at T1(Pearson correlation 0.2634, p < 0.01). This variable was not included in best subset regression analysis, reason unspecified. Neither of the above variables correlated with any psychological outcomes at T2&3. “Experience of the funeral and mourning rituals … failed to influence bereavement outcome” – no data provided to support this	Only including nuclear families, religiously homogenous sample, dropout characteristics not identified, unclear reporting of outcomes, qualitative data collected not reported
Mitima-Verloop et al. (2019) *Netherlands*	**Study design:** observational prospective cohort quantitative survey**Population:** individuals bereaved within the last 6 months recruited via routinely administered customer satisfaction survey of a funeral service company **Intervention:** perception of the funeral using *Funeral Evaluation Questionnaire (FEQ)*, grief rituals using derivative of *Bereavement Activities Questionnaire (BAQ)* **Outcome measures:** grief using *Traumatic Grief Inventory self-report version (TGI-SR)*, positive and negative feelings using *Positive and Negative Affect Scale (PANAS)*, impairment in functioning using *Work and Social Adjustment Scale (WSAS)* at T1 (time of first survey) and T2 (3 years later)	552 at T1, 289 at T2	At T1: Mean age 58.9 years58.5% femaleNationality and religion collected at T2: 97.6% Dutch (without migration background), 2.4% other29.6% Christian, 16.7% Spiritual, 50.2% no religion, 3.5% other	Participants perceived the funeral as contributing to processing their loss (agreed with the statement “The way in which the period around the funeral was organized, was important in processing the loss” “a lot” to “very much” 75.9% at T1 and 70.2% at T2) with a high mean item score (M=4.07, SD=1.07 at T1 and M=3.92, SD=1.11 at T2).Positive association between general evaluation of funeral and positive affect at T1 (r-0.21, p < 0.001) and funeral director evaluation and positive affect (r=0.13, p = 0.003)Hierarchical regression analysis with grief and general evaluation of funeral and funeral director at T1 scores predicting grief scores at T2 was significant (F = 248.82, p < 0.001). However grief at T1 explained a unique proportion in variance in grief at T2 (β=0.696, p < 0.001) but not the other two variables (p = 0.596 and p = 0.283 respectively)	Recruitment from satisfaction survey, culturally homogenous sample, T1 to T2 dropouts significantly demographically different to T2 participants, FEQ designed for this study and not validated
Saler & Skolnick (1992) *U.S.*	**Study design:** observational cross-sectional quantitative survey**Population:** adults who had experienced the death of one parent before the age of 18 years, and were now aged between 20 and 50 years recruited from public notices**Intervention:** children’s participation in various mourning activities using *The Mourning Behaviour Checklist (MBC)* **Outcome measures:** parental attitudes and behaviour using *Parental Bonding Instrument (PBI),* depressive symptomology using *The Center for Epidemiological Studies Depression Scale (CES-D),* depressive experience using *The Depressive Experiences Questionnaire (DEQ)*	90	Mean age 32.2 years58% female43% Jewish, 26% Catholic, 14% Protestant, 7% no religion92% white, 8% Asian/black/Hispanic/other	MBC was the only statistically significant variable in multiple regression analyses determining contribution to CES-D (β=0.2876, p = 0.0133 when using PBI raw score and β=0.3212, p = 0.0067 when using PBI parenting style scores), MBC was significantly associated with higher Self-Criticism scores on DEQ (β=0.23, p≤0.05) i.e. those who reported less opportunity for participation in mourning activities had higher rates of depressive symptomology and were more prone to self-criticism	Ethnically homogenous sample, retrospective self-reporting of mourning activities, breakdown of specific question contributions not reported
Schaal et al. (2010) *Rwanda*	**Study design:** observational cross-sectional quantitative survey**Population:** widows (who had not remarried) and orphans (lost one or more parents) over 18 years old who had experienced the Rwanda genocide in 1994**Intervention:** self-reported funeral attendance**Outcome measures:** Prolonged Grief Disorder diagnostic status and symptom severity using *PG-13*, Post-traumatic stress disorder symptoms using the *PTSD Symptom Scale Interview (PSS-I)*	400	87.7% femaleMean age 37.18 years61% Catholic, 23.3% Protestant, 4% Islamic, 2% Adventist, 6% other, 3.8% no religion	Multiple regression analysis with grief score as dependent variable showed funeral attendance did not significantly contribute to the severity of prolonged grief reactions (BPGD-score -1.14, B SEPGD-score 0.68, bPGD-score -0.06. p-values not given).	Losses due to violence may not be generalisable, predominantly female sample
Weller et al. (1988) *U.S.*	**Study design:** observational cross-sectional quantitative survey**Population:** bereaved children agreed 6-12 years with a IQ ≥70 and no chronic incapacitating medical or psychiatric illnesses present and their surviving parent, and the following applied: at least one parent had been employed the majority of the time in the 2 years preceding death; no chronic incapacitating illness in either parent had been present in the 2 years preceding the death (other than that associated with the deceased parent's death); no family member had received inpatient or outpatient psychiatric treatment in those previous 2 years; the surviving parent was able to complete the questionnaires and be interviewed; children of divorcedparents had to have had frequent visitation with both parents; parental death was not caused by suicide or homicide. Local obituary section used, funeral home director/clergy contacted to discuss appropriateness of contacting family, if appropriate family contacted**Intervention:** children’s participation in funeral activities using *Death Related Behaviour Questionnaire – Child/Adult Form***Outcome measures:** presence/absence of psychiatric diagnosis using *The Diagnostic Interview for Children and Adolescents (DICA-C/DICA-P),* grief experiences using *The Grief Interview – Child/Parent Form*	38 children, 26 parents	Children: 47% maleParents: 73% female87% white, 8%H Hispanic, 5% black	“T tests were used to determine whether children’s participation in … funeral activities was associated with [depression or anxiety] symptomology … The two groups did not differ significantly in depressive, anxiety or other psychiatric symptomology as rated by the child or parent.” No interpretive statistics provided to support this	Very strict inclusion criteria, ethnically homogenous sample, predominantly female sample, interpretive statistics not presented
Zisook & DeVaul (1983) *U.S.*	**Study design:** observational cross-sectional quantitative survey**Population:** friends and colleagues of the authors who had lost a relative or close friend**Intervention:** self-reported funeral attendance**Outcome measures:** unresolved grief using *Unresolved Grief Index*	211	62% female65% white, 17% black, 11% Mexican American, 7% otherMean age 36.5 years47% Protestant, 26%Catholic, 13% Jewish, 13% other/none	Participants with “definitely unresolved grief” (score of ≥6 on Unresolved Grief Scale) were less likely to have attended the funeral (p < 0.05)	Recruitment method, unvalidated questions measuring outcome, interpretive statistics not specified
Aksoz-Efe et al. (2018) *Turkey*	**Study design:** qualitative phenomenological semi-structured interviews **Population:** Turkish women who had experienced a death loss and early traumatic experience recruited by a college’s psychiatry department and psychological counselling guidance programme **Aim:** examining the definition and meaning of grief, examine possible participant perceived connections between cultural bound death-related rituals and beliefs and their grief	8	Age 25–59 yearsAll Muslim	*Metaphors of loss*Destruction in the lives of the bereaved, pattern and wholeness of the family corrupted, irreversibility and inevitability of death*Funeral rituals*Funeral rituals most commonly involved visits to the deceased’s house (the funeral house), which continue from 7–40 days after death to meet the physical, psychological and social needs of the bereaved. Specific rituals include praying from the Quran for 7 days, not turning on TV for 7 days, the 7th, 40th and 52nd days (i.e., visits for prayers), Mevlid (i.e., spiritual poems about the prophet Muhammed), turning on the light of the room in which the person died, and taking some souvenirs from the deceased. Importance of seeing the body of the deceased. If the deceased’s body has to wait in the home, it is covered with a white sheet called *kefen* which can be opened just to leave the face and sometimes the hand out for the bereaved to see the deceased for the last time*Rituals in relation to control, age, socioeconomic status and grief*Rituals either deemed helpful or unhelpful depending on participants’ perceived *control* over rituals: when religious practice based on own will, experienced relief; when imposed, felt guilty and distressed. Younger, higher socioeconomic status women experienced more control as they were employed and therefore had the ability to be physically away from home. The specific elements of ritual that emerged as most critically related to participants’ grief were religious beliefs, condolence visits, talking about the deceased, and cultural expectations of when grief “should” end. Praying, thinking that their loved ones were good people and believing that they will be in Heaven were helpful. Participants also reported unhelpful aspects related to religious beliefs andpractices. In some cases, when participants were questioning their beliefs after the loss, others were perceived as putting pressure on them about not losing their faith. Because of the seemingly imposed behaviours of others, some of the participants described feeling guilty and distressed for not fulfilling their religious duties. The imposed religious expectations were viewed as new and additional burdens by the participants. After a while, usually the first week, participants described feeling stressed about visits and crowds, and need to be alone and have their own time to focus on feelings and thoughts. Participants were unable to experience their grief due to “preaching” messages of others. Concern about judgement among those with no personal space or control over their grief. All participants reported being particularly concerned and stressed when other people were around. After 30–40 days, people expected them to start acting normally. Participants described being encouraged to stop grieving, continue with their lives and focus on daily life responsibilities. Again, helpful or unhelpful depending on sense of control	Variable time after loss
Chan et al. (2005) *Hong Kong*	**Study design:** retrospective qualitative analysis of individual bereavement counselling interviews**Population:** bereaved former clients of a community-based bereavement centre aged over 18 years who received counselling within the last 2 years, not recruited for this study, former counselling recordings analysed **Aim:** explore the bereavement process of Chinese persons in Hong Kong and examine the influence of the Chinese culture on the experience of the bereaved	10 counselling sessions analysed; themes then compared to 42 other transcripts	78.85% female44.23% no religion, 25% traditional ancestor worship, 9.62% Buddhism, 21.15% Christianity	*Meaning making*Cause of death categorised in terms of traditional beliefs (fate, karma, fate clashes among family members, feng shui, evil spirit). Presence of family at moment of death appraised positively and negatively by equal numbers. In Chinese culture, there is a strong belief in the afterlife. Most had a positive sense of the destination of the deceased in the afterlife. Participants mentioned that their relationship with the deceased (*yuan*) would be continued. Two thirds of participants perceived that it was good to participate in the funeral to have the final change to say goodbye to the deceased. The remaining third harboured negative feelings, largely because they had not been able to participate. During a Taoist funeral ritual, the lay priest (*Nahm Mouh*) chants and performs sacrificial litanies. Similar rituals by Buddhist nuns and monks. Viewed positively mostly, expect when costly or contrasting with bereaved person’s belief system. Ritual viewing of body before final covering of coffin had some negative memories. “Soul-flag” and “purifying wash” (symbolically washing a piece of the deceased’s clothing) viewed positively by half, but negatively by half as they did not have a son who would usually carry out rituals*Bond continuation*Continuing the bond with the deceased anchored in traditional Chinese beliefs. Continuation of relationship initiated by either the deceased (feeling, hearing or seeing the deceased) or by bereaved (talking to a photo of the deceased, visiting graveyard or cemetery)	Therapeutic interviews used so not aiming to answer research question directly
Nesteruk (2018) *U.S.*	**Study design:** retrospective qualitative analysis of semi-structured interviews from a larger study**Population:** immigrants in the United States aged 45 and older who arrived as young adults (aged 18-30 years) recruited through senior centres, residential facilities, personal contacts of the author and snowball sampling**Aim:** advance understanding of long-term immigrants’ coping with death and bereavement in the transnational context	56	Mean age 6476.8% femaleOriginated from 31 different countries	*Caregiving in transnational families*Participants described a sense of duty to either provide care directly or financially contribute to care from a distance. Sense of obligation and guilt when reflecting on aging loved ones in countries of origin. Participants described feelings of distress at their inability to attend a loved one’s funeral. *Coping with loss and transnational grieving*Participants who were able to attend funerals in their countries of origin felt that being with their loved ones and participating in rituals associated with death, such as making funeral preparations, sharing meals, praying, and attending services, provided them with a sense of belonging and comfort. Grieving alone in adoptive country was challenging due to the lack of social support. Being surrounded by familiar rituals and being able to share one’s emotions with others who are also grieving helps one process the loss, not feel alone, and facilitate coping. Other participants emphasized that being remote from the death of a loved one provided an emotional barrier from the familial upheaval and cultural drama that is often associated with the end-of-life rituals in their countries of originIncreasingly, technological advances in communication enable immigrants to go online to witness the services of their loved ones, see family, and virtually participate in the cultural rituals associated with death. Although these technological advancements may not be commonplace in every situation, they do represent an opportunity for immigrants to virtually cross large distances and borders, and to be present at the funeral services for a loved one.*Family discontinuity and anticipatory grief as mitigating factors*Immigrants lose physical contact, support, and the presence of family members which in time may lead to a discontinuity of family relationships. Over time this leads to a greater self-reliance and the ability to manage change and loss without family support. Loss may be mitigated by years of separation. Family of procreation and work duties demanded attention and distracted from loss.	Part of a large study not aimed at examining bereavement experiences in detail therefore not aiming to directly answer research question
Pang and Lam (2002) *Hong Kong*	**Study design:** qualitative semi-structured interviews **Population:** widowers who had taken part in either Chinese or Christian death rituals**Aim:** report widowers’ bereavement experiences and how the widowers go through the bereavement process by the performance of Chinese and Christian death rituals	4	Age 37–50 years2 Christian, 2 no religion	*Funeral rituals*Personalised elements of funeral rituals provided meaning and eased grief, provided a chance of catharsis to restore emotional stability. Benefited from console given by the funeral participants whose attendance confirmed the value of the decease. However, low attendance at funeral was interpreted as a lack of social support which contributed to feeling worse. *Postfuneral rituals*With the performance of post funeral death rituals, beliefs were reinforced and a continuing bond with the deceased was established	Recruitment process not specified, reporting of individual cases as opposed to working out commonality and themes
Silverman (1987) *U.S.*	**Study design:** qualitative interviews**Population:** college-age women who had lost a parent to death recruited from a nearby graduate professional school**Aim:** To examine female children’s reactions to the death of a parent	18	11.1% blackMost respondents from upper middle-class families	*Coping with death*With one exception, all their families had traditional funerals. For the older women, seeing the body seemed to be important. Participant who did not felt it may have made grieving easier. Some women felt that their participation in the funeral made a difference and that the funeral brought them together as a family. Other women, especially several who were teenagers at the time, felt resentful of the way they were included and, in the long run, seemed to have more difficulty coping. Those who were younger at the time of death seemed reassured by being included among the mourners and knowing what to expect. For those that were already teenagers, they wanted to be involved in a more adult fashion in the decision making, but not in making the actual decisions. Although data support the idea that participation in the funeral and associated religious ritual is important, in the long run, parental responsiveness from the very outset was most critical, in the eyes of these women, to how they managed. Coping by trying to carry on as normal – by being busy they could avoid a confrontation with their new reality. However, difficulties with concentration and study. Other people’s concerns seemed trivial and frivolous. Increased isolation from peers, learning to keep their feelings to themselves. *The bereavement process*All participants continue to deal with the death, no reporting of closure. As adults, they needed to renegotiate their relationships to their parents alive and dead. Acknowledgement of what they have missed and what the death meant to the surviving parent.	Recruitment process unclear, information about participants limited
Vandercreek & Mottram (2009) *U.S.*	**Study design:** qualitative semi-structured interviews**Population:** suicide bereaved female individuals aged over 21 years who suffered the suicide of a family member at least 2 years in the past, recruited through suicide support groups (first 10 volunteers accepted) **Aim:** explore the function of survivor’s personal religion, support from family and friends, and established religious communities during bereavement	10	All Caucasian9 Christian, 1 Buddhist7 considered religion “very important”, 1 “somewhat important”, 2 “not important”	*The functions of the survivor’s personal religion*Bereavement process led over time to a new, clearer religious purpose in life. *The function of religious support from family and friends*Many families and friends expressed support through sympathy cards or verbally. Limited help as implied not willing to help wrestle with bereavement concerns. Meaningful support from those chosen by the bereaved or those who had also lost a child by suicide. *The functions of established religious communities*All survivors held a funeral and many people attended. The survivors tended to interpret the attendance of these friends, acquaintances, and concerned strangers as religious support.A survivor found the expression of support at the funeral helpful even at the time of the interview many years after the suicide	Religiously homogenous sample

## Results

**Figure 1.**
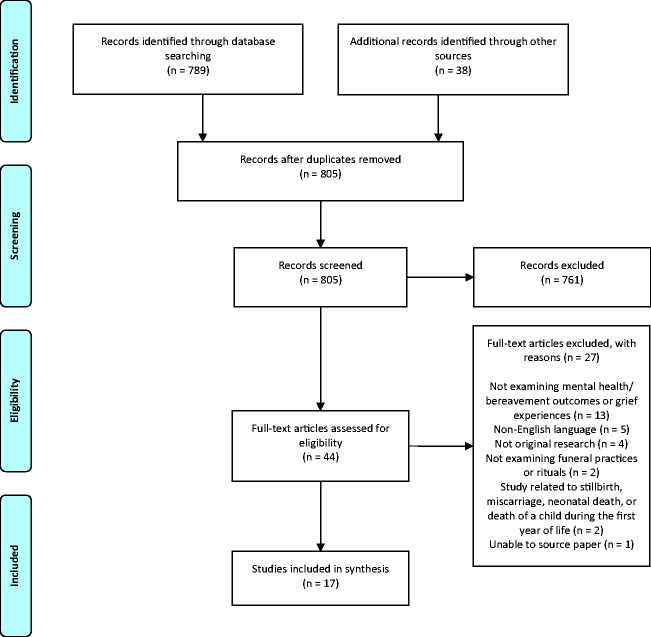
PRISMA Flow Diagram.

All searches were performed on 24th April 2020. We identified 789 articles from MEDLINE and PsycINFO searches and 38 articles from other sources. After deduplication, 805 articles remained for screening. Searches of references from commentaries/narrative reviews identified were performed (n = 3) but resulted in no further relevant articles. Forty-four articles underwent full-text review, with 17 included in the analysis (Figure 1; Table 2).

Eleven observational and six qualitative studies were identified.Most were conducted in the U.S. (Bolton & Camp, 1987; Doka, 1985; Fristad, et al. , 2001; Gamino et al., 2000; Grabowski & Frantz, 1993; Nesteruk, 2018; Saler & Skolnick, 1992; Silverman, 1987; Vandercreek & Mottram, 2009; Weller et al., 1988; Zisook & DeVaul, 1983) in White Christian populations.Other country settings were Hong Kong (Chan et al., 2005; Pang & Lam, 2002), Australia (Kissane et al., 1997), Netherlands (Mitima-Verloop et al., 2019), Rwanda (Schaal et al., 2010) and Turkey (Aksoz-Efe et al., 2018). The included articles, published between 1983 and 2019, were of variable quality (Tables 3 and 4). Participant numbers ranged from 50 to 552 in the observational studies.

**Table 3.** Quantitative Study Quality—Assessed Using the Quality Assessment Tool for Observational Cohort and Cross-Sectional Studies.

Author (year)	1	2	3	4	5	6	7	8	9	10	11	12	13	14	Total
Bolton & Camp (1987)	Yes	No	CD	Yes	No	No	Yes	Yes	No	No	Yes	No	NA	No	5
Doka (1985)	Yes	Yes	Yes	Yes	No	No	Yes	NA	No	No	Yes	No	NA	No	6
Fristad et al. (2001)	Yes	No	No	No	No	No	Yes	NA	Yes	Yes	Yes	No	Yes	No	6
Gamino et al. (2000)	Yes	No	CD	No	No	No	Yes	NA	No	NA	Yes	CD	NA	No	3
Grabowski & Frantz (1993)	Yes	No	CD	No	No	No	CD	NA	No	NA	Yes	No	NA	No	2
Kissane et al. (1997)	Yes	Yes	Yes	Yes	No	No	Yes	NA	No	NA	Yes	No	No	Yes	7
Mitima-Verloop et al. (2019)	Yes	Yes	No	Yes	No	No	Yes	Yes	No	Yes	Yes	No	No	Yes	8
Saler & Skolnick (1992)	Yes	Yes	Yes	No	No	No	Yes	Yes	No	No	Yes	No	NA	Yes	7
Schaal et al. (2010)	Yes	Yes	Yes	Yes	No	No	Yes	Yes	No	NA	Yes	No	NA	Yes	8
Weller et al. (1988)	Yes	Yes	No	Yes	No	No	No	Yes	No	No	Yes	No	NA	No	5
Zisook & DeVaul (1983)	Yes	No	CD	No	No	No	No	Yes	No	No	No	No	NA	No	2

CD, cannot determine; NA, not applicable.

*Questions:*.

1. Was the research question or objective in this paper clearly stated?

2. Was the study population clearly specified and defined?

3. Was the participation rate of eligible persons at least 50%?

4. Were all the subjects selected or recruited from the same or similar populations (including the same time period)? Were inclusion and exclusion criteria for being in the study prespecified and applied uniformly to all participants?

5. Was a sample size justification, power description, or variance and effect estimates provided?

6. For the analyses in this paper, were the exposure(s) of interest measured prior to the outcome(s) being measured?

7. Was the timeframe sufficient so that one could reasonably expect to see an association between exposure and outcome if it existed?

8. For exposures that can vary in amount or level, did the study examine different levels of the exposure as related to the outcome (e.g., categories of exposure, or exposure measured as continuous variable)?

9. Were the exposure measures (independent variables) clearly defined, valid, reliable, and implemented consistently across all study participants?

10. Was the exposure(s) assessed more than once over time?

11. Were the outcome measures (dependent variables) clearly defined, valid, reliable, and implemented consistently across all study participants?

12. Were the outcome assessors blinded to the exposure status of participants?

13. Was loss to follow-up after baseline 20% or less?

14. Were key potential confounding variables measured and adjusted statistically for their impact on the relationship between exposure(s) and outcome(s)?

**Table 4.** Qualitative Study Quality—Assessed Using Critical Appraisal Skill Programme Qualitative Checklist.

Author (year)	1	2	3	4	5	6	7	8	9	10	Overall quality
Aksoz-Efe et al. (2018)	Yes	Yes	Yes	Yes	Yes	No	No	Yes	Yes	Discussed with relevant literature, identified areas for further research	Good
Chan et al. (2005)	Yes	Yes	Yes	Yes	No	No	No	No	Yes	Discussed with relevant literature, identified areas for further research, discussed implications for practitioners	Moderate
Nesteruk (2018)	Yes	Yes	Yes	Yes	Yes	Yes	No	Yes	Yes	Discussed with relevant literature, identified areas for further research, discussed implications for practitioners	Good
Pang & Lam (2002)	Yes	Yes	No	Can’t tell	Can’t tell	No	No	No	Yes	Discussed with relevant literature, discussed implications for practitioners	Low
Silverman (1987)	Yes	Yes	No	Yes	No	No	No	No	Yes	Discussed with relevant literature, identified areas for further research	Moderate
Vandercreek & Mottram (2009)	Yes	Yes	Yes	Yes	Yes	No	No	Yes	Yes	Discussed with relevant literature, identified areas for further research	Good

*Questions:*.

1. Was there a clear statement of the aims of the research?

2. Is a qualitative methodology appropriate?

3. Was the research design appropriate to address the aims of the research?

4. Was the recruitment strategy appropriate to the aims of the research?

5. Was the data collected in a way that addressed the issue?

6. Has the relationship between researcher and participants been adequately considered?

7. Have ethical issues been taken into consideration?

8. Was the data analysis sufficiently rigorous?

9. Is there a clear statement of the findings?

10. How valuable is the research?

Overall, evidence of the effect of funeral participation on mental health or bereavement outcomes was inconclusive. Five observational studies found funerals were associated with significant benefits to bereaved participants; the positive impact on grief experiences are described further in the six qualitative studies. However, the remaining six observational studies found no measurable associations.

*Involvement in planning a funeral* was not associated with grief adjustment at one year in a study of 50 white, middle-to-upper class Americans. In another component of this mixed-methods study, 57% felt planning a funeral helped with their grief, 28% were unsure, and 15% felt it was unhelpful and viewed funeral planning as a routine duty (Doka, 1985). Gamino et al. (2000) reported that bereaved individuals who participated in planning the funeral reported significantly lower depersonalisation (p = 0.001) and social isolation (p = 0.05) than those who did not. In a qualitative study of immigrants in the U.S., participants who were able to attend funerals in their countries of origin reported gaining a sense of belonging and comfort from participating in funeral preparations with their loved ones (Nesteruk, 2018).

*Funeral attendance* was associated with less unresolved grief in one U.S. study (Zisook & DeVaul, 1983), with participants who were found to have “definitely unresolved grief” significantly less likely to have attended the funeral of their relative or close friend (p < 0.05). These findings should be interpreted with caution as this study had a number of methodological limitations: there was no clear definition of the study population, no application of inclusion or exclusion criteria, and no description of methods used for data analysis; the measure used for unresolved grief was also unvalidated and its development was not described in detail. In a study of widows who had experienced the 1994 Rwandan genocide, funeral attendance did not significantly contribute to the severity of prolonged grief reactions in a multiple regression analysis (Schaal et al., 2010). This is clearly a unique setting and these results may not be generalisable to other scenarios. A U.S. study found no significant differences in grief intensity between those who did and did not attend a funeral in Latino and Anglo-American samples. In the Latino sample, there was no significant difference in grief intensity between those who had and had not participated in a *novena*, a post-funeral practice involving prayer and support that generally takes place in the home of the bereaved (Grabowski & Frantz, 1993). In contrast, in qualitative studies, participants perceived funeral participation positively as a chance to say goodbye (Chan et al., 2005), and those unable to participate due to geographical distance reported distress (Nesteruk, 2018; Pang & Lam, 2002):

*“Everybody is there together and they are there for each other to give comfort. Here, we are the only ones. [All we can do] is just cry, that’s it. So, it is hard.”* (Nesteruk, 2018, p. 9)

A participant in Nesteruk’s study of immigrants in America described her experience of virtually attending her father’s funeral in India:

*“We were on Skype and whatever was going on—I was there. The whole night, sitting online, praying and seeing my daddy until the last moment when they took him away. So, I felt that I was there with him all the time.”* (Nesteruk, 2018, p. 10)

*Post-death rituals:* In Bolton and Camp’s (1987) study, the number of self-reported pre-, during-, and post-funeral rituals performed after a death was not associated with grief adjustment, although no interpretative statistical data are provided to support this statement. An Australian longitudinal study found that 6 weeks after a death, viewing the body was associated with fewer depressive symptoms (p < 0.001) and less intense grief (p < 0.01), and saying goodbye as wished with better social adjustment (p < 0.01), but these associations were not evident at 6 months or 13 months (Kissane et al., 1997).

In a qualitative study of Muslim participants in Turkey, traditional post-death rituals were perceived as helpful or unhelpful in the grieving process, with their reported helpfulness dependent on participants’ sense of control over and involvement in them. In particular, the practice of not leaving the bereaved alone was perceived by participants who retained a sense of personal space and control as supportive, while others perceived the same ritual to be difficult or even disturbing. Participants who felt a lack of control were generally older and financially dependent on others (Aksoz-Efe et al., 2018).

In two studies in Hong Kong, rituals were generally viewed positively and provided meaning, easing grief and reinforcing bonds with the deceased (Chan et al., 2005; Pang & Lam, 2002). The exception was a group of widows describing their pain at the Taoist ritual of ‘breaking the comb’, performed before covering the coffin to symbolize the end of the marriage between the bereaved and deceased (Chan et al., 2005).

*Funeral experience:* In a recent longitudinal study from the Netherlands, funerals were perceived as contributing to processing the loss by >70% of participants at 6 months (T1) and 3 years (T2) post bereavement, and a positive evaluation of the funeral and funeral director was associated with positive affect at T1 (p < 0.001). A regression analysis including grief, general evaluation of the funeral and evaluation of the funeral director at T1 predicted grief at T2 (p < 0.001), however when looking at individual components of the model, only grief at T1 explained a unique proportion in variance of grief at T2 (p < 0.001). Of note, participants were recruited via a funeral service’s satisfaction survey and there was a high dropout rate from T1 to T2 (552 to 289 participants), with significant differences between those who withdrew and those who did not (Mitima-Verloop et al., 2019).

In a U.S. study, mourners who described a funeral as ‘comforting’ reported significantly less overall grief (p = 0.01), social isolation (p = 0.005), despair (p = 0.01), anger/hostility (p = 0.02) and guilt (p = 0.05), although the nature of these associations (and in particular what causal mechanisms might be at play) is unclear. Adverse events during the funeral service – for example conflicts among survivors, discrepancies between the wishes of the deceased and the bereaved, and problems with cremation – were associated with higher overall grief (p = 0.05) and other poor outcomes including somatisation (p = 0.001), loss of control (p = 0.02), and depersonalisation (p = 0.05) (Gamino et al., 2000).

In qualitative research, low funeral attendance was perceived by relatives as a lack of social support (Pang & Lam, 2002), while high funeral attendance was supportive, and remained helpful for years afterwards:

*“Even now, I think back to that day and it gives me strength because there were so many people who supported us.’’* (Vandercreek & Mottram, 2009, p. 751)

In Hong Kong, some negative memories were reported of the ritual viewing of the body before the final covering of the coffin:

*“Whenever I close my eyes, the image of my father pops up obsessively. His eyes were half opened, and so was his mouth. His face was white like a cement wall, contrasting with the lips with lip-stick as if he was bleeding. I cannot wash away this awful memory.”* (Chan et al., 2005, p. 16)

*Children’s bereavement outcomes and experiences:* A U.S. study of 38 children with restrictive inclusion criteria (see Table 2) found no association between a child’s funeral participation and depression or anxiety as reported by parent or child 2 months post-death, although no specific data were provided to support this statement (Weller et al., 1988). Fristad et al. (2001) studied parent-bereaved children in the U.S., and found less opportunity to participate in funeral-related activities was associated with higher rates of depressive symptomatology at 13- and 25- months post-loss. This was, however, in an analysis that was not pre-specified: nearly all children whose families had visitations, funerals and burials attended these, therefore comparison was instead made between participants whose families did and did not have a visitation. In the same study, children were asked open-ended questions regarding what they did and didn’t like about the funeral and associated rituals. The most common responses regarding what they liked related to aspects of the ritual (e.g. music such as the deceased’s favourite song, flowers, prayers, poems, or the act of putting something into the casket), the support of others, and the eulogy. Children also appreciated the finality of death represented by the funeral and burial. Aspects of the funeral children did not like included the behaviour of others (e.g. friends, relatives); seeing the deceased or their physical appearance (e.g., *“did not like their smell and their lips sealed”*, Fristad et al., 2001, p. 7); not liking the preacher or minister (*“the minister did not know my father”*, p. 8) or the lowering of the casket during the burial.

In a study of adults bereaved as a child (Saler & Skolnick, 1992), having less opportunity to participate in mourning activities as a child was associated with higher rates of depressive symptomology and likelihood of being prone to self-criticism. In qualitative research, however, U.S. college-age women who were teenagers at the time of bereavement had varying views: some resented being involved in the funeral and reported more difficulty coping, whilst others felt their participation brought the family closer (Silverman, 1987).

## Discussion

This review is the first to synthesise evidence regarding the effect of funeral practices on bereaved friends’ and relatives’ mental health and bereavement outcomes. We found no systematic reviews in this area and the only quantitative studies were observational in nature, examining associations between funeral practices and bereavement/mental health outcomes rather than establishing causal relationships, which would require randomised designs unethical in this context. Across observational studies of variable quality, some found benefits associated with funeral participation while others did not. However, the qualitative research identified provides useful additional insight: for both adults and children, the benefit of after-death rituals including funerals depends on the ability of the bereaved to shape those rituals and say goodbye in a way which is meaningful for them, and on whether the funeral demonstrates social support for the bereaved.

In the context of COVID-19, these findings suggest that restrictions to funeral practices do not necessarily entail poor outcomes or experiences for the bereaved: it is not the number of attendees or even the type of funeral which determines how supportive it is, but rather how meaningful the occasion is, and how connected it helps mourners feel. Similarly, a research study published since our searches were conducted found no association between type nor elaborateness of cremation service and levels of grief (Birrell et al., 2020), although these participants chose their funeral arrangements as opposed to having restrictions imposed. The review findings also highlight the crucial role played by funeral directors and officiants in helping the bereaved to create funerals which are personal, meaningful and expressive of collective grief and support despite the current restrictions associated with COVID-19. Creating a funeral in this context requires sensitivity, creativity and skill, especially since it may be harder to create an emotional connection with the bereaved when arranging a funeral service virtually; however, there are resources to support this process (Table 5). Our findings suggest that ‘template’ services where the funeral officiant takes a leading role might be less appropriate than personalised services in which families take the lead and the officiant facilitates, supporting people to create and perform their own rituals (Kuyvenhoven, 2020). The latter approach also reflects the role funeral providers play in bereavement support: in an Australian survey, their support was perceived as very/quite helpful by 91.30% of respondents, second only to family support (Aoun et al., 2018). With social support from family and friends limited during the pandemic, funeral providers’ and officiants’ part in bereavement support is particularly relevant.

**Table 5:** . Resources for Meaningful Funerals During COVID-19.

Idea for how to include others (Kuyvenhoven, 2020; Virtual Funeral Collective, 2020)	- Live-stream funerals – welcome and thank those joining remotely - Record services to watch retrospectively - Hold ‘rolling funerals’ with a series of virtual meetings to remember the deceased - Hold alternative ceremonies at the time of the funeral e.g. in a families’ garden. Neighbours may wish to witness from across rooftops and fences. - Share lists of songs, photos, and stories which remind the bereaved of the person they have lost - Read out personal tributes or play voice recordings sent by people who can’t attend - Use a candle, flowers or cards with a photo and personalised message to represent people who can’t attend - Offer alternative rituals or commemorative activities e.g. playing a meaningful song, lighting a candle, writing a letter to the person who has died, planting seeds and baking a favourite cake - Host online memorials and notes of remembrance Families may wish to organise an online reception where invited friends and families can raise a glass, wear the person’s favourite colour and/or share memories
Planning a meaningful funeral	For adults: https://quakersocialaction.org.uk/we-can-help/helping-funerals/down-earth/coronavirus-organising-meaningful-funeral For children: https://www.winstonswish.org/coronavirus-funerals-alternative-goodbyes/
Memorializing	http://www.suddendeath.org/covid-19-bereavement/covid-19-advice-on-memorialising
Information and resources on a wide range of topics to support funeral directors, officiants and the bereaved	Virtual Funeral Collective (2020). Death, Grief and Funerals in the COVID Age: https://covidwhitepaper.com/download
Mourning collective loss	Evans A., ter Kuile C. and Williams I (2020) This Too Shall Pass: Mourning collective loss in the time of Covid-19: https://www.collectivepsychology.org/wp-content/uploads/2020/04/This-Too-Shall-Pass.pdf

Emerging evidence suggests that during the pandemic, the bereaved and the funeral providers and officiants supporting them have shown remarkable resilience, finding novel alternatives to usual practices and traditions. A U.K. report published since our literature searches describes positive experiences of smaller funerals during the pandemic, with those able to attend appreciating their intimate and personal nature (Bear et al., 2020). When physical funeral attendance isn’t possible, virtual attendance has been facilitated through live-streaming or other means, including integrating the presence of those who are absent in other ways (Table 5) (Bear et al., 2020; Walter et al., 2011). In non-academic literature published during the pandemic, religious leaders and attendees have praised live-streamed funeral services for widening access – for example, enabling Muslim women to experience the burial – and enabling private emotional expression not possible at public ceremonies (Wood, 2020). Our review did not find any literature examining whether or how virtual attendance impacts mental health or bereavement outcomes, although one participant’s experiences of virtual funeral attendance were described positively by Nesteruk (2018). Comparing the grief and bereavement outcomes of virtual attendance to both physical attendance and not being able to attend a funeral at all is a recommended avenue for exploration.

We found that in qualitative research, a sense of control was a key determinant of whether participants identified funeral practices and rituals as helpful or unhelpful. Given the social restrictions and economic uncertainty during the COVID-19 pandemic, many people will be experiencing a sense of lack of control over their lives. This may be particularly true in Black, Asian and Minority Ethnic (BAME) groups which are underrepresented in research in this area, overrepresented in lower socioeconomic groups and have a markedly higher mortality risk from COVID-19 (Razaq et al., 2020). Calls for governments to provide financial support for funeral costs to communities disproportionately affected by the pandemic are therefore appropriate (Bear et al., 2020). To further support vulnerable communities, we recommend engaging with community and religious leaders to provide culturally sensitive information and support regarding local bereavement services and funerals.

Only one of the studies we identified explicitly examined ethnic, cultural, or religious differences in funeral practices and mental health, grief, or bereavement outcomes (Grabowski & Frantz, 1993). Funeral practices vary widely between groups along all of these axes and different faith and cultural groups will be affected to varying degrees by current restrictions (Bear et al., 2020; Uzell, 2018). Washing the body of the deceased, for example – an important ritual in Islam, Judaism and Sikhism – is currently restricted in the U.K. to either only being carried out in full personal protective equipment with supervision, or not at all (Public Health England, 2020). COVID-19 has also disrupted the Jewish ritual of *shiva,* seven days of intense mourning in which the community provides meals, prayer and comfort, as well as has the Irish tradition of the wake and the Baptist repast that follows a funeral (Schuck et al., 2020). While every individual will be affected differently by restrictions to their mourning process, these losses may be a source of communal as well as individual distress (Bear et al., 2020). An improved understanding of the impact of restrictions on funeral practices on different communities’ bereavement outcomes and experiences could help funeral officiants in adapting services and inform the advice bereavement service providers give to families. Technology is being used to accommodate gatherings of friends and family for prayer, recitation of the rosary or a wake, to coordinate virtual *shiva* visits, and to hold the nine night ceremonies traditional across the Caribbean diaspora (Schuck et al., 2020; White, 2020); however the acceptability and impact of these adaptations is not yet known.

The quantitative studies identified used a range of grief, social adjustment, and psychological symptomology outcome measures. There was, however, a notable lack of research on how these outcomes may impact on subsequent psychiatric and psychological diagnosis, treatment and service use, be that primary care, specialist bereavement services, or psychiatric care. Clinical correlates should be explored in future research to enable service providers to anticipate demand and allocate resources accordingly.

## Limitations

This review excluded non-English language reports and may therefore have omitted relevant articles. As with any review, there is a risk of publication bias impacting the outcome of literature searches. The evidence identified was published from 1983 to 2019; during this time there have been considerable changes in how funerals are designed and conducted. In particular, in recent years, celebrant-led, person-centred services have grown in popularity. These changes should be taken into account when considering the external validity of our findings, and future research should reflect the diversity of the current funeral landscape. While existing evidence on funeral practices and bereavement outcomes provides useful indicative guidance, its generalisability to the unprecedented funeral restrictions currently in place are unknown.

## Conclusion

The prevalence of mental health conditions is likely to increase during and immediately after the COVID-19 pandemic (Nobles et al., 2020), with those who have lost a family member at particular risk of psychiatric distress (Chew et al., 2020). The social support available to those who are bereaved is limited, and social isolation is known to exacerbate psychological morbidity in bereavement (Selman et al., 2020).Based on this review, it is unclear how funeral restrictions will contribute to this on a population level. However, qualitative research highlights the importance of meaningful and supportive funerals for the bereaved; enabling relatives to achieve a sense of control and social support despite current restrictions is crucial, especially among BAME communities most vulnerable at this time. As well as access to bereavement support and sign-posting to specialist services, palliative care and bereavement teams should provide locally-relevant information regarding the creation of meaningful, culturally appropriate funerals. As people continue to find new ways to grieve and commemorate their loved one, the impact of these alternative modalities should be explored. Becoming bereaved during COVID-19 presents challenges at every stage of the funeral process, from planning to post-funeral rituals and memorialisation. Understanding the personal and public health effects of this will take time and sensitive, methodologically robust research.

## Supplemental Material

sj-pdf-1-ome-10.1177_0030222820941296 - Supplemental material for How do Funeral Practices impact Bereaved Relatives' Mental Health, Grief and Bereavement? A Mixed Methods Review with Implications for COVID-19 Click here for additional data file. Supplemental material, sj-pdf-1-ome-10.1177_0030222820941296 for How do Funeral Practices impact Bereaved Relatives' Mental Health, Grief and Bereavement? A Mixed Methods Review with Implications for COVID-19 by Alexander Burrell and Lucy E. Selman in OMEGA–Journal of Death and Dying
